# Application Value of Serum TK1 and PCDGF, CYFRA21-1, NSE, and CEA plus Enhanced CT Scan in the Diagnosis of Nonsmall Cell Lung Cancer and Chemotherapy Monitoring

**DOI:** 10.1155/2022/8800787

**Published:** 2022-03-25

**Authors:** Xiang He, Ming Wang

**Affiliations:** ^1^Department of Diagnostic CT, Cangzhou Central Hospital, Cangzhou, Hebei, China; ^2^Department of Radiology, Cangzhou Central Hospital, Cangzhou, Hebei, China

## Abstract

**Objective:**

To assess the application value of serum thymidine kinase 1 (TK1) and PC cell-derived growth factor (PCDGF), cytokeratin 19 fragment 21-1 (CYFRA21-1), neuron-specific enolase (NSE), and carcinoembryonic antigen (CEA) plus enhanced CT scan in the diagnosis of nonsmall cell lung cancer (NSCLC) and chemotherapy monitoring.

**Methods:**

Between April 2019 and April 2021, 30 patients with NSCLC assessed for eligibility treated in our institution were included in the experimental group, and 30 healthy individuals screened out from physical examinations were recruited in the control group. The chemotherapy regimens included gemcitabine plus cisplatin, pemetrexed disodium plus cisplatin, and vinorelbine plus cisplatin. The application value of serum TK1, PCDGF, CYFRA21-1, NSE, CEA, and enhanced CT scan in the diagnosis and chemotherapy monitoring of NSCLC was analyzed.

**Results:**

Before treatment, the eligible patients had significantly higher serum levels of TK1, PCDGF, CYFRA21-1, NSE, and CEA than those of the healthy individuals included (*P* < 0.05). Clinical efficacy was categorized into good and poor, and the good efficacy included complete response and partial response, with the poor efficacy including stable disease and progressive disease. Patients with good clinical efficacy had lower levels of serum TK1, PCDGF, CYFRA21-1, NSE, and CEA than those with poor efficacy (*P* < 0.05). Joint detection showed a larger area under the curve (AUC) (0.900; 95%CI, 0.812-0.988), a higher sensitivity, and a superior detection outcome to the stand-alone detection (*P* < 0.05). Diagnostic results were similar between joint detection and pathological examination (*P* > 0.05).

**Conclusion:**

The application of serum TK1, PCDGF, CYFRA21-1, NSE, and CEA assay plus enhanced CT scan shows high sensitivity and diagnostic accuracy in the diagnosis and chemotherapy monitoring of nonsmall cell lung cancer and thus provides a diagnostic reference basis.

## 1. Introduction

Lung cancer is a common malignant tumor, with high morbidity and mortality rates, posing a serious threat to patient's health and life safety [1]. According to statistics [2], the morbidity and mortality rates of lung cancer in men occupy the first place among all malignancies, and in women, the morbidity ranks third and the mortality ranks second. Clinical data show that nonsmall cell lung cancer (NSCLC) is associated with approximately 85% of lung cancer, and many cases have progressed to the advanced stage at the time of diagnosis due to the insidious symptoms and missed diagnosis. The 5-year survival of NSCLC is merely 11%-15% [3]. Thus, an urgent need exists to achieve effective early diagnosis and treatment of NSCLC patients. Diagnostic indices used clinically for NSCLC include cytokeratin fragment 19 antigen 21-1 (CYFRA21-1), neuron-specific enolase (NSE), and carcinoembryonic antigen (CEA), which, however, were found from relevant studies with modest sensitivity and specificity to meet clinical needs [4]. Given the insufficiency of single tumor marker assays in the diagnosis of lung cancer, combination with other detection methods is entailed to enhance the diagnostic accuracy [5, 6]. Thymidine kinase 1 (TK1), an enzyme that catalyzes the phosphorylation of thymidine to generate thymidine monophosphate, is strongly associated with DNA synthesis and cell proliferation and has demonstrated high diagnostic value in the NSCLC [7, 8]. PC cell-derived growth factor (PCDGF), a member of the growth regulator family, promotes tumorigenesis and development by binding to receptors and is expressed abnormally among lung cancer patients in particular [9, 10]. With the improvement of medical technology, CT scans are considered highly valuable in tumor diagnosis, but for a robust diagnostic accuracy, the combination of relevant tumor markers is also desired. Accordingly, this study was conducted to assess the application value of serum TK1, PCDGF, CYFRA21-1, NSE, and CEA plus enhanced CT scan in the diagnosis of NSCLC and chemotherapy monitoring.

## 2. Materials and Methods

### 2.1. Participants and Grouping

From April 2019 to April 2021, 30 eligible patients with NSCLC treated in our institution were recruited as the experimental group, and 30 healthy individuals screened out from physical examinations were included in the control group. The clinical baseline features of the eligible patients (19 males and 11 females, aged between 48 and 76 years, mean age of (54.35 ± 5.61) years, 9 cases in TNM stage I-II and 21 cases in stage III-IV, 12 cases of squamous cell carcinoma, 6 cases of adenocarcinoma, 8 cases of small cell carcinoma, and 4 cases of large cell carcinoma) were comparable with those of the healthy individuals (18 males and 12 females, aged between 46-75 years, mean age of (55.29 ± 6.34)) (*P* > 0.05).

### 2.2. Inclusion and Exclusion Criteria

Inclusion criteria: (1) all patients who were diagnosed with NSCLC confirmed by imaging and histopathology; (2) with no chemotherapy, radiotherapy, or surgery before randomization; (3) the study was approved by the hospital ethics committee, and patients and family members had provided written informed consent.

Exclusion criteria: (1) patients with serious heart, liver, kidney, and other functional abnormalities; (2) with other serious infectious diseases; (3) with other malignant tumors; (4) with mental illness.

### 2.3. Methods

#### 2.3.1. Chemotherapy

The eligible patients were all primary cases.

Gemcitabine (Manufacturer: Qilu Pharmaceutical (Hainan) Co., Ltd.; State Drug Quantifier: H20113286; Specification: 1.0 g) plus cisplatin (Manufacturer: Yunnan Botanical Pharmaceutical Co., Ltd.; State Drug Registration: H53021740; Specification: 2 ml: 10 mg) regimen: gemcitabine 1250 mg/m^2^, d1-8 and cisplatin 75 mg/m^2^, used in 2 d. Pemetrexed disodium (Manufacturer: Qilu Pharmaceutical Co., Ltd.; State Pharmacopoeia: H20060672; specification: 0.2 g) plus cisplatin regimen: pemetrexed disodium 500 mg/m^2^, and cisplatin 75 mg/m^2^, used in 2 d. Vinorelbine (Manufacturer: Jiangsu Hengrui Pharmaceutical Co., Ltd.; State Drug Quantifier: H20061234; specification: 20 mg) plus cisplatin: vinorelbine 25 mg/m^2^, d1-8, cisplatin 75 mg/m^2^, used in 2 d. One treatment cycle consists of 21 d. Serum tumor markers determination and systemic assessment were performed after every 2 cycles of chemotherapy.

#### 2.3.2. Sample Collection

After fasting more than 10 h, 5 ml of morning venous blood was collected from all participants and centrifuged at a radius of 15 cm and 2500 r/min for 10 min to obtain the serum which was then stored at -20°C.

#### 2.3.3. Measurement of Serum Markers

Serum TK1, PCDGF determination: the serum TK1 level was determined by immunoradiometric analysis, and serum PCDGF level was determined by ELISA using double-antibody sandwich enzyme-linked immunosorbent assay. Serum CYFRA21-1, NSE, CEA determination: the CEA level was determined using the immunoradiometric assay, the CYFRA21-1 level was determined using the immunoradiometric assay, and the NSE level was determined using electrochemiluminescence. All assays were performed per the standards of the kit instructions.

#### 2.3.4. Enhanced CT Scan

A Siemens 64-row spiral CT machine was used to perform the contrast-enhanced CT scan, with the scanning conditions of 180 mA and 120 kv, a scanning area from the entrance of the thorax to the diaphragm plane, a layer thickness of 5 mm, and a scanning scope of 8 cm. The contrast agent was iohexol, 80-100 ml, and was administered through intravenous bolus injection at a rate of 3 ml/s using a high-pressure syringe. Multiphase scans were performed at 10 s, 15 s, 25 s, 50 s, 65 s, 120 s, 160 s, and 200 s after the injection.

### 2.4. Outcome

(1) Comparison of serum TK1, PCDGF, CYFRA21-1, NSE, and CEA levels before treatment between the two groups; (2) clinical efficacy of patients in the experimental group after chemotherapy. Clinical efficacy was evaluated as per RECIST criteria [11]: complete response (CR): all lesions disappeared, no new lesions appeared, tumor markers returned to normal and the above conditions were maintained for at least 4 weeks. Partial response (PR): the sum of the longest diameter of the tumor was reduced by ≥30% or more, and the above condition was maintained for at least 4 weeks. Stable disease (SD): the sum of the longest tumor diameters decreased by less than 30% or increased by less than 20%. Progressive disease (PD): the sum of the longest diameter of the tumor increased by ≥20% or new lesions appeared. Clinical efficacy was categorized into good and poor, and the good efficacy included complete response and partial response, with the poor efficacy including stable disease and progressive disease. (3) Comparison of serum TK1, PCDGF, CYFRA21-1, NSE, and CEA levels after treatment between the two groups. (4) Analysis of the correlation between posttreatment serum TK1, PCDGF, CYFRA21-1, NSE, and CEA levels in the eligible patients and the efficacy. (5) Analysis of the diagnostic value of serum TK1, PCDGF, CYFRA21-1, NSE, CEA levels, enhanced CT scan, and joint detection.

### 2.5. Statistical Analysis

SPSS20.0 was used for data analyses, and GraphPad Prism 7 (GraphPad Software, San Diego, USA) was used to visualize the data into matching images. Count data were expressed as (*n* (%)) and were subject to the chi-square test. The measurement data were expressed as ($\bar{x}\pm s$) and processed using the *t*-test. Differences were considered statistically significant when *P* < 0.05.

## 3. Results

### 3.1. Serum TK1, PCDGF, CYFRA21-1, NSE, and CEA before Treatment

Before treatment, the serum levels of TK1, PCDGF, CYFRA21-1, NSE, and CEA of the eligible patients were higher than those of the healthy individuals included (*P* < 0.05) (Table 1).

### 3.2. Clinical Efficacy

**Figure 1:**
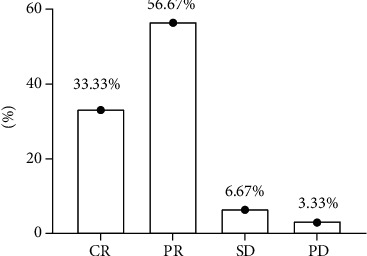
Clinical efficacy of the eligible patients. Note: the abscissa indicates the clinical efficacy, and the ordinate indicates the percentage, %. Among the eligible patients, there were 10 cases of CR, 17 cases of PR, 2 cases of SD, and one case of PD.

The CR, PR, SD, and PD of the eligible patients were 33.33%, 56.67%, 6.67%, and 3.33%, respectively (Figure 1).

### 3.3. Serum Levels of TK1, PCDGF, CYFRA21-1, NSE, and CEA in Patients with Different Clinical Efficacy

Eligible patients with good clinical efficacy showed lower levels of serum TK1, PCDGF, CYFRA21-1, NSE, and CEA versus those with poor efficacy (*P* < 0.05) (Table 2).

### 3.4. AUC of Detection Approach

**Figure 2:**
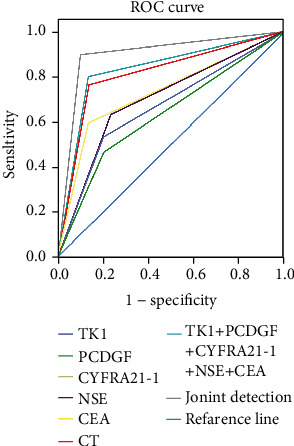
Comparison of AUC of single and joint detection.

The joint detection using serum TK1, PCDGF, CYFRA21-1, NSE, CEA, and enhanced CT scan yielded a larger AUC versus stand-alone detection (Figure 2).

### 3.5. Detection Outcomes

Joint detection of serum TK1, PCDGF, CYFRA21-1, NSE, and CEA, plus enhanced CT scan outperformed stand-alone detection using either the serum tumor markers or enhanced CT scan (*P* < 0.05) (Table 3).

### 3.6. Sensitivity and Specificity

A significantly higher sensitivity was obtained in the joint detection when compared with stand-alone detection (Table 4).

### 3.7. Diagnostic Results

Diagnostic results were similar between joint detection and pathological examination (*P* > 0.05) (Table 5).

## 4. Discussion

Nonsmall cell lung cancer is a type of lung malignancy originating from the bronchial mucosa, bronchial glands, and alveolar epithelium and is classified into adenocarcinoma, squamous cell carcinoma, adenosquamous carcinoma, large cell carcinoma, and sarcomatoid subtypes as per histopathology [12]. The development of NSCLC is strongly associated with environmental factors and genes, such as age, genetics, immune and nutritional status, bad living habits, and environmental pollution. The insidiousness of early-stage symptoms usually results in an undesirable treatment timing [13, 14], which necessitates the significance of early diagnosis and treatment for NSCLC patients. Tumor markers are simple and noninvasive in the diagnosis of lung cancer, with great merit in the diagnosis of tumors, disease monitoring, and efficacy assessment [15]. Serum NSE is an acidic protease specific to neurons and neuroendocrine, a specific marker of neuroendocrine tumors [16, 17]. The origin of the NSCLC from neuroendocrine cells results in a key role of serum NSE in diagnosing NSCLC. CEA is an acidic glycoprotein with a human embryonic antigenic determinant cluster and a broad-spectrum tumor marker, which is widely used in differential diagnosis, disease monitoring, and efficacy determination of tumors [18]. CYFR21-1 is a filamentous substance that constitutes the cytoskeleton and is abundantly released during cell carcinogenesis to get involved in cell proliferation and metastasis [19]. Results of the present study showed lower levels of pretreatment CYFRA21-1, NSE, and CEA of the eligible patients versus healthy individuals, indicating that the diagnostic value of CYFRA21-1, NSE, and CEA in NSCLC. Research results by Wang et al. [20] revealed a mediocre sensitivity and specificity of the above three indices and proposed that joint detection may potentiate the diagnostic accuracy.

Serum TK1 is a cell cycle-dependent marker that is closely related to cell proliferation as a kinase expressed in the cytoplasm [21]. It is a highly potent IA marker of cell proliferation with an essential role in systemic organ tumorigenesis and development, and its expression level indicates the degree of active cell proliferation [22]. PCDGF is involved in tumor cell adhesion, cell proliferation, angiogenesis, and extracellular matrix degradation through stimulation of mitogen-activated protein kinase signaling pathway, phosphatidylinositol 3-kinase signaling pathway, and local adhesion kinase signaling pathway to promote tumor cell infiltration and metastasis, where its high expression levels are associated with tumor development [23]. Here, the eligible patients showed significantly higher levels of TK1 and PCDGF versus healthy individuals (*P* < 0.05), which was consistent with the research results by Chen et al. [24]. They revealed that the highly expressed TK1 and PCDGF levels were associated with the heavy release of TK1 from cancer tissue cells into the peripheral circulation and the regulatory role of PCDGF in tumor immunity. In the present study, patients with better clinical efficacy had lower serum levels of TK1, PCDGF, CYFRA21-1, NSE, and CEA versus those with poor clinical efficacy (*P* < 0.05), indicating that the above indices demonstrate great potential in chemotherapy monitoring to provide a reference basis. CT scan is a major instrument for the diagnosis of NSCLC, which features excellent spatial and density resolution of lung tissues, improves the accuracy of target area contours, avoids missed imaging of target areas, and reduces radiation complications of normal tissues and organs, with high accuracy in the diagnosis of lung cancer. This study found that the sensitivity of the combined assay was significantly higher than that of the stand-alone detection, and there was no statistical difference (*P* > 0.05) between the joint detection results and the pathological results, indicating a high diagnostic value of the combined assay.

To sum up, the application of serum TK1, PCDGF, CYFRA21-1, NSE, and CEA assay plus enhanced CT scan shows high sensitivity and diagnostic accuracy in the diagnosis and chemotherapy monitoring of nonsmall cell lung cancer and thus provides a diagnostic reference basis.

## Figures and Tables

**Table 1:** Comparison of serum TK1, PCDGF, CYFRA21-1, NSE, and CEA before treatment.

Indices	Control group	Experimental group	*t*	*P*
TK1 (pmol/L)	0.72 ± 0.31	7.49 ± 1.55	23.459	<0.001
PCDGF (ng/ml)	9.11 ± 0.57	18.86 ± 3.19	16.480	<0.001
CYFRA21-1 (ng/ml)	2.10 ± 0.42	7.56 ± 1.24	22.843	<0.001
NSE (ng/ml)	10.45 ± 1.78	19.63 ± 7.28	6.709	<0.001
CEA (ng/ml)	2.97 ± 1.03	29.47 ± 7.35	19.557	<0.001

**Table 2:** Correlation of serum TK1, PCDGF, CYFRA21-1, NSE, and CEA with clinical efficacy.

Indices	Good efficacy (*n* = 27)	Poor efficacy (*n* = 3)	*t*	*P*
TK1 (pmol/L)	1.47 ± 0.64	5.39 ± 1.20	9.266	<0.001
PCDGF (ng/ml)	10.58 ± 1.16	17.41 ± 3.03	8.131	<0.001
CYFRA21-1 (ng/ml)	2.37 ± 0.52	7.04 ± 1.13	13.116	<0.001
NSE (ng/ml)	12.65 ± 1.29	16.51 ± 2.05	4.669	<0.001
CEA (ng/ml)	4.32 ± 1.06	10.34 ± 3.66	7.099	<0.001

**Table 3:** Comparison of detection outcomes.

				95% confidence interval	
Variables	Area	Standard error^a^	Progressive sig.^B^	Lower limit	Upper limit
TK1	0.667	0.071	0.027	0.528	0.806
PCDGF	0.633	0.072	0.076	0.491	0.775
CYFRA21-1	0.700	0.069	0.008	0.565	0.835
NSE	0.700	0.069	0.008	0.565	0.835
CEA	0.733	0.066	0.002	0.603	0.864
CT	0.817	0.058	0.000	0.703	0.931
TK1+PCDGF+CYFRA21-1+NSE+CEA	0.833	0.056	0.000	0.724	0.943
Joint detection	0.900	0.045	0.000	0.812	0.988

**Table 4:** Comparison of sensitivity and specificity between stand-alone detection and joint detection.

Variables	Positive if greater than or equal to^a^	Sensitivity	1-Specificity
TK1	-1.0000	1.000	1.000
	0.5000	0.533	0.200
	2.0000	0.000	0.000

PCDGF	-1.0000	1.000	1.000
	0.5000	0.467	0.200
	2.0000	0.000	0.000

CYFRA21-1	-1.0000	1.000	1.000
	0.5000	0.633	0.233
	2.0000	0.000	0.000

NSE	1.0000	1.000	1.000
	0.5000	0.633	0.233
	2.0000	0.000	0.000

CEA	1.0000	1.000	1.000
	0.5000	0.633	0.133
	2.0000	0.000	0.000

CT	1.0000	1.000	1.000
	0.5000	0.600	0.133
	2.0000	0.000	0.000

TK1+PCDGF+CYFRA21-1+NSE+CEA	1.0000	1.000	1.000
	0.5000	0.767	0.133
	2.0000	0.000	0.000

Joint detection	1.0000	1.000	1.000
	0.5000	0.800	0.100
	2.0000	0.000	0.000

**Table 5:** Comparison of diagnostic results between joint detection and pathological examination.

Diagnostic results	*n*	Diagnosed	Misdiagnosed and missed diagnosed	Diagnostic rates
Pathological examination	30	30 (100.00)	0.00% (0/30)	30 (100.00)
Joint detection results	30	29 (96.67)	1 (3.33)	29 (96.67)
*x* ^2^				1.017
*P*				0.313

## Data Availability

The datasets used during the present study are available from the corresponding author upon reasonable request.

## References

[1] Fan L., Chen H., Teng J. (2018). Evaluation of serum-paired miRNA ratios for early diagnosis of non-small cell lung cancer using quantum dot-based suspension array. *Journal of Nanomaterials*.

[2] Liu X., Tang C., Song X. (2020). Clinical value of CTLA4-associated microRNAs combined with inflammatory factors in the diagnosis of non-small cell lung cancer. *Annals of clinical biochemistry*.

[3] Kumar S., Sharawat S. K., Ali A. (2020). Identification of differentially expressed circulating serum microRNA for the diagnosis and prognosis of Indian non-small cell lung cancer patients. *Current Problems in Cancer*.

[4] Udumyan R., Montgomery S., Fang F. (2020). Beta-Blocker use and lung cancer mortality in a nationwide cohort study of patients with primary non-small cell lung cancer. *Cancer epidemiology biomarkers and prevention*.

[5] Gemine R. E., Ghosal R., Collier G. (2019). Longitudinal study to assess impact of smoking at diagnosis and quitting on 1-year survival for people with non-small cell lung cancer. *Lung cancer: Journal of the International Association for the Study of Lung Cancer*.

[6] De Marinis F., Barberis M., Barbieri V. (2019). Diagnosis and first-line treatment of non-small cell lung cancer in the era of novel immunotherapy: recommendations for clinical practice. *Expert review of respiratory medicine*.

[7] Kamel L. M., Atef D. M., Mackawy A. M., Shalaby S. M., Abdelraheim N. (2019). Circulating long non-coding RNA GAS5 and SOX2OT as potential biomarkers for diagnosis and prognosis of non-small cell lung cancer. *Biotechnology and Applied Biochemistry*.

[8] Tribukait B. (2020). Early prediction of pathologic response to neoadjuvant treatment of breast cancer: use of a cell-loss metric based on serum thymidine kinase 1 and tumour volume. *BMC Cancer*.

[9] Kumar J. K., Holmgren S., Levedahl K. H., Höglund M., Venge P., Eriksson S. (2020). AroCell TK 210 ELISA for determination of TK1 protein: age-related reference ranges and comparison with other TK1 assays. *BioTechniques*.

[10] Ren H., Hu Y., Xie T., Jin C., Hu Y., Yang B. (2019). Effect of gefitinib on serum EGFR and CYFRA21-1 in patients with advanced non-small cell lung cancer.. *Oncology letters*.

[11] Yang H., Bao J., Huo D. (2021). Au doped poly-thionine and poly-m-Cresol purple: Synthesis and their application in simultaneously electrochemical detection of two lung cancer markers CEA and CYFRA21-1. *Analytical Chemistry*.

[12] Xia Y. M., Xia M., Zhao Y., Li M. Y., Ou X., Gao W. W. (2021). Photocatalytic electrochemical sensor based on three-dimensional graphene nanocomposites for the ultrasensitive detection of CYFRA21-1 gene. *Microchemical Journal: Devoted to the Application of Microtechniques in all Branches of Science*.

[13] Cheng J., Hu K., Liu Q., Liu Y., Yang H., Kong J. (2021). Electrochemical ultrasensitive detection of CYFRA21-1 using Ti3C2Tx-MXene as enhancer and covalent organic frameworks as labels. *Analytical and bioanalytical chemistry*.

[14] Weagel E. G., Mejía J., Kovtun R. (2018). TK1 membrane expression may play a role in the invasion potential of A549 lung cancer cells. *Journal of Cancer Therapy*.

[15] Weagel E. G., Burrup W., Kovtun R. (2018). Membrane expression of thymidine kinase 1 and potential clinical relevance in lung, breast, and colorectal malignancies. *Cancer Cell International*.

[16] Ge Y. L., Liu C. H., Wang M. H. (2019). High serum neuron-specific enolase (NSE) level firstly ignored as normal reaction in a small cell lung cancer patient: a case report and literature review. *Clinical laboratory*.

[17] Oya Y., Yoshida T., Uemura T., Murakami Y., Inaba Y., Hida T. (2018). Serum ProGRP and NSE levels predicting small cell lung cancer transformation in a patient with ALK rearrangement-positive non-small cell lung cancer: a case report. *Oncology letters*.

[18] Jin W., Lei Z., Xu S. (2021). Genetic Mutation Analysis in Small Cell Lung Cancer by a Novel NGS-Based Targeted Resequencing Gene Panel and Relation with Clinical Features. *BioMed Research International*.

[19] Cao B., Wang P., Gu L., Liu J. (2021). Use of four genes in exosomes as biomarkers for the identification of lung adenocarcinoma and lung squamous cell carcinoma. *Oncology letters*.

[20] Wang X., Liao X., Zhang B. (2021). An electrochemical immunosensor for the detection of carcinoembryonic antigen based on Au/g-C3N4 NSs-modified electrode and CuCo/CNC as signal tag. *Microchimica Acta*.

[21] Clevers M. R., Kastelijn E. A., Peters B. J., Kelder H., Schramel F. M. (2021). Evaluation of serum biomarker CEA and Ca-125 as immunotherapy response predictors in metastatic non-small cell lung cancer. *Anticancer Research: International Journal of Cancer Research and Treatment*.

[22] Zheng W., Zhao J., Tao Y. (2018). MicroRNA‑21: A promising biomarker for the prognosis and diagnosis of non‑small cell lung cancer (Review). *Oncology letters*.

[23] Chen Y., Mathy N. W., Lu H. (2018). The role of VEGF in the diagnosis and treatment of malignant pleural effusion in patients with non‑small cell lung cancer (Review). *Molecular medicine reports*.

[24] Chen G., Zheng Z., Li J. (2021). Long non-coding RNA PITPNA-AS1 silencing suppresses proliferation, metastasis and epithelial-mesenchymal transition in non-small cell lung cancer cells by targeting microRNA-32-5p. *Molecular medicine reports*.

